# Analysis of Nanomaterials on Biological and Environmental Systems and New Analytical Methods for Improved Detection

**DOI:** 10.3390/ijms23116331

**Published:** 2022-06-06

**Authors:** Sarah Reagen, Julia Xiaojun Zhao

**Affiliations:** Department of Chemistry, University of North Dakota, Grand Forks, ND 58202, USA; sarah.reagen@und.edu

**Keywords:** nanoparticles, engineered nanomaterials, nanotoxicity, in vitro, in vivo, analytical chemistry, method standardization

## Abstract

The advancing field of nanoscience has produced lower mass, smaller size, and expanded chemical composition nanoparticles over recent years. These new nanoparticles have challenged traditional analytical methods of qualification and quantification. Such advancements in nanoparticles and nanomaterials have captured the attention of toxicologists with concerns regarding the environment and human health impacts. Given that nanoparticles are only limited by size (1–100 nm), their chemical and physical characteristics can drastically change and thus alter their overall nanotoxicity in unpredictable ways. A significant limitation to the development of nanomaterials is that traditional regulatory and scientific methods used to assess the biological and environmental toxicity of chemicals do not generally apply to the assessment of nanomaterials. Significant research effort has been initiated, but much more is still needed to develop new and improved analytical measurement methods for detecting and quantitating nanomaterials in biological and environmental systems.

## 1. Introduction

**Figure 1:**
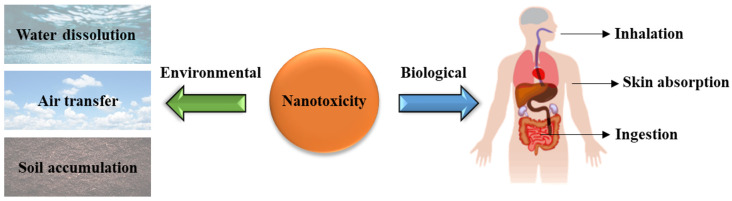
Nanotoxicity exposures for environmental and biological matrices.

Nanoscience has consistently been a developing and advancing field with a great diversity of applications in medicine, energy, electronics, biotechnology, materials, etc. [1]. Engineered nanomaterials (ENMs) and nanoparticles (NPs) are simply defined as materials with at least one dimension of 1–100 nm in size [2]. This definition means that their various chemical and physical properties allow them to be altered and changed to perform their targeted functions and tasks [2,3,4]. Additionally, due to their vast diversity in multiple research fields, nanoparticles have now been incorporated into common everyday products such as food preservatives, cosmetics, clothes, etc. [4]. This constant unseen contact with nanoparticles has promoted the field of nanotoxicology to study the greater impact these ENMs have on both biological and environmental systems (Figure 1) [5]. However, due to limitations in analytical instrumentation and analytical test methods directly applicable to measuring ENMs in the environmental and biological matrices, nanotoxicity remains an underdeveloped field as it struggles to keep up with the advancing research and development of nanoparticles and nanoparticle-based materials actively being developed [6,7].

One differentiating characteristic is the size of the nanoparticles, which can make them chemically different from larger particles and bulk materials (e.g., diffusivity across cell membranes) [2,4,5,6,7,8]. Additional unique biological cell interactions come in the form of ENMs surface charge, with anionic and neutral ENMs generally having lower toxicity than cationic materials. ENMs surface charges may also have an additional influence on the particles’ overall shape, and the shape of ENMs can alter cell membranes as well, thus heavily influencing the cellular uptake mechanism [5,8,9]. Surface coatings of ENMs can alter their toxicity by providing additional electrostatic forces, molecular adhesion, and atomic layer deposition, contributing to cell death [9]. Furthermore, the elemental composition of ENMs contributes to their overall toxicity to both biological and environmental systems [10]. Such elements can range from transition metals (gold, silver, copper, iron, etc.) to non-metals (silica, carbon). They can greatly alter the previously listed size, morphology, coating, and physical and chemical properties.

NPs and ENMs are primarily introduced into the environment through consumer products [11]. This problem has many arising concerns due to low detection concentrations, usually ng/L, and the current limits of detection of analytical instruments [12]. NPs can also be integrated into the human body in a multitude of ways, but most commonly through inhalation, ingestion, and skin absorption, while environmental exposure is usually through the air, water, and soil integration [4]. For biological matrices, ENMs can impact the mitochondrial function of cells and produce reactive oxidative species (ROS). The analytical measurement of mitochondrial function, damage, and ROS levels in biological systems remains a primary tool in assessing toxicity [13]. ROS levels greatly impact cell metabolism as they are natural byproducts of cell metabolism and contribute to cell survival, death, signaling, inflammation, and differentiation [13]. An imbalance of ROS leads to disrupted redox homeostasis in cells, which ultimately interferes with the cell’s overall function in relation to DNA/RNA breakage, membrane destruction, protein carbonylation, and other means [14]. However, ROS compounds have been looked at previously as an alternative to chemotherapy for cancer treatment [15]. Radical compounds such as superoxide (O_2_^•−^), hydroxyl (HO^•^), hydroperoxyl (HO_2_^•^), peroxyl (RO_2_^•^), alkoxyl (RO^•^), carbon dioxide (CO_2_^•−^), carbonate (CO_3_^•−^) and singlet oxygen (^1^O_2_) are involved in key cell reactions that revolve around signaling and homeostasis processes [13,14,15]. However, high levels of ROS compounds can result in oxidative damage to healthy cells and interfere with cell metabolism. ROS accumulation contributes to normal cells turning into cancer cells [16,17]. The introduction of NPs into biological systems can interfere with ROS generation in several ways depending on the characteristics of the NPs [13].

With the field of nanoscience and nanotoxicity expanding, this perspective aims to investigate what analytical techniques are used in toxicology assessments to effectively measure ENMs and NPs toxicity. Furthermore, this perspective will also include advancing analytical techniques to better detect and evaluate ENMs and NPs in biological and environmental matrices and what future methods could be introduced to better detect ENMs and NPs toxicity. However, there is no current federal or state legislation in the United States specifically for the regulation and testing of nanomaterials. Regulatorily, there are agencies such as the intergovernmental Organization for Economic Co-operation and Development (OECD), the American Industrial Hygiene Association (AIHA), and the US Environmental Protection Agency (USEPA) that investigate ENMs and their greater impact on human health and the environment as well as regulate analytical methods of testing ENMs. Given the increasing amount of ENMs being integrated into consumer and industrial products, the OECD has identified a greater need than ever to have accurate testing methods since the potential risks and impacts of nanomaterials are not well developed. To date, the OECD has documented over 780 studies on the specific physiochemical properties of nanomaterials that contribute to plant/animal toxicity as well as ecotoxicity [18]. Additionally, with the evolving field of ENMs, the OECD has continued to modify current methods and promote new ones as a means of keeping up with the advancing technology around ENMs [18].

## 2. Current Methods and Concerns for In Vitro Nanotoxicity Determination

Due to the variety of factors that impact ENMs and NPs toxicity, as previously mentioned, there is not a singular method for accurate detection of ENMs and NPs toxicity. Rather, there are several methods that are commonly used in conjunction to help identify the characteristics of ENMs and NPs and their overall toxicity [19]. Dynamic light scattering (DLS) is most commonly used to determine hydrodynamic particle size, and zeta potential (also determined by the DLS instrument) determines particle surface charge. At the same time, methods such as scanning electron microscopy (SEM) and transmission electron microscopy (TEM) allow for the visual detection of ENMs and NPs that can then be measured for their size distribution. Although these methods give insight into the characteristics of the ENMs and NPs, they do not give toxicity analyses. For this, researchers turn to in vitro and in vivo examinations.

To examine in vitro toxicity first, one standard measurement technique to measure ENMs and NPs toxicity in vitro studies is MTT (3-[4,5-dimethylthiazole-2-yl]-2,5-diphenyltetrazolium bromide) assays, which assess the cells’ mitochondrial function by detecting mitochondrial dehydrogenase through an enzymatic reduction. Another standard measurement technique for toxicity determination is examining ROS formation within the cells, which indicates oxidative stress and interference in cell function. In order to measure intracellular ROS, a fluorescent ROS indicator is typically utilized. When in the presence of ROS, this indicator will chemically change and thus yield a different fluorescent signal. This signal can be observed through fluorescence spectroscopy [20,21] or through confocal microscopy [22]. The most common in vitro assays are summarized in Table 1 [23].

However, these two methods are not completely accurate means of determining toxicity. For example, an interesting study was performed by Dönmez Güngüneş et al., where three different kinds of NPs (Fe_3_O_4_, fullerenes (C_60_) and single-walled carbon nanotubes (SWCNT)) were tested in two different cell lines (human periodontal ligament fibroblasts (hPDLF) and mouse dermal fibroblast (mDF)) [24]. Although the MTT assay and ROS analyses of the three different kinds of NPs showed that the hPDLF cells, compared to the mDF cells, were more susceptible to all three NPs (showing higher ROS levels and larger MTT decrease in cell viability), Dönmez Güngüneş et al. utilized a relatively newer analytical method known as xCELLigence where a gold microelectrode is labeled with an antigen to which the test cells are exposed. The current between the gold and reference electrode will increase as the cells neutralize the antigen blocking the signal on the gold surface, which allows for real-time kinetic measurement of cell health and behavior. Dönmez Güngüneş et al. found that although the human cells were more susceptible to the NPs in terms of raw cell viability in the MTT assays and mitochondrial failure due to ROS formation, the internal mechanism of the cells remained unchanged with all three types of NPs tested. However, the mouse cells showed internal failure as most of the cells no longer performed as they should, an indication of some toxic effects within the cells. Without this third analysis in xCELLigence, one could have concluded that the mouse cells were relatively unaffected by the NPs solely based on the MTT and ROS analyses without knowing the true impact on the intercellular mechanisms that were impacted to a larger degree compared to the human cells.

## 3. Current Methods and Concerns for In Vivo Nanotoxicity Determination

Furthermore, there are concerns around unintended particle accumulation in organs and thus induced toxicity for in vivo studies. The potentially hazardous NPs are directly or indirectly introduced into living organisms with measurements of toxicology endpoints. Several nanoparticles, such as gold-based and other metal-based NPs, display toxic effects and organ accumulation. However, the toxicity pathways are not fully understood [25,26]. The most common organs tested for and impacted by NPs accumulation are the liver, heart, kidney, spleen, lungs, intestine, and stomach. The liver and organs with high blood flow are the most unintended accumulation sites [27,28]. Which organs are impacted more depends on the elemental composition and size of the NPs [29]. For instance, carbon-based NPs show the most unintended accumulation in the liver [30]. However, smaller carbon-based particles less than 20 nm, such as quantum dots (QDs), showed increased accumulation in the brain parenchyma [31]. The QDs can pass through the BBB pathway and through the trigeminal nerve or olfactory epithelium, which can cause additional problems when investigating in vivo toxicity [32,33]. However, despite the accumulation of carbon-based particles in organs, due to their chemical makeup, carbon-based NPs typically display little to no significant increase in toxicity when examined in vivo [34]; however, some toxic effects have been recorded [35].

Silica-based NPs show similar low toxicity when accumulated in organs compared to carbon-based NPs, although some uterine metabolic issues have been discovered in mice [36]. Silica NPs accumulate the most in the liver, lungs, and spleen [37], with some kidney accumulation also being observed [38]. Histological studies of silica-based NPs showed no ill effects in organs when the NPs were cleared within a few months [39,40]. For NPs composed of less harmful chemicals such as carbon and silica, their size plays a much greater role in their toxicity in addition to their chemical composition. Generally, smaller particles are more toxic due to their size, allowing them to better interact with cellular components such as proteins, fatty acids, and nucleic acids [41]. However, larger silica NPs have also been shown to possess greater toxicity than smaller silica NPs [40]. Polymer- and metal-based NPs with low clearance rates generally showed the greatest toxicity and organ accumulation [42,43] and sometimes contained greater metabolic disturbance [44].

However, several limitations remain for the in vivo analysis of NPs organ accumulation and long-term toxicity. For some studies, a lack of macrophage uptake and blood circulation suggests the need for better assays [45]. Additionally, in vivo studies with animals do not necessarily carry over to human studies as many nanoparticles never reach their intended site and are cleared from the bloodstream quickly [46], adding to the difficulty of detecting in vivo toxicity and attributing it to NPs. Furthermore, most in vivo studies examine toxicity via week- or month-long analyses. Year-long analyses are rarely examined in research primarily due to time constraints despite being informative and essential [47]. Nonetheless, these are all considerable parameters when examining in vivo nanotoxicity, with several current methods needing improved testing parameters and animal models for more accurate toxicity assessments, especially when examining the complexity of human health [48]. The most commonly used in vivo analysis methods are listed in Table 2 [23].

To specifically measure nanotoxicity in vivo, several factors are taken into consideration. Immune system response compounds such as globulin, TNF-alpha, and KC-GRO are typically analyzed to determine toxicity. However, correlation is not necessarily causation, as simply analyzing these markers after introducing ENMs is not an accurate means of analyzing toxicity. A far more exact means of determining ENMs toxicity in vivo is by utilizing ICP-MS and microwave digestion, where tissue and organic samples are prepared via microwave digestion before being subjected to ICP-MS. This is a more accurate way of confirming ENMs integration and concentration within key organs, as shown in a study by Weaver et al. [49]. This is because there needs to be a way to confirm that the amount of ENMs or NPs injected into the animal remained in the organs and did not pass through the bloodstream without interactions. Unfortunately, ICP-MS is limited to metal-based NPs and cannot be used for NPs that are, for example, polymer-based. For these NPs, it is much more difficult to determine their concentration and integration in vivo accurately. Therefore, methods previously mentioned (such as fluorescence, bioluminescence, microscopy, and spectroscopy) are utilized as accurately as possible [50,51]. Additionally, smaller-sized NPs concentrations tend to be more difficult to determine [52]; with the importance of concentration control for in vitro and in vivo, it is vital to accurately determine the biological target tissue concentration for nanotoxicity tests [53]. However, despite the extensive means to confirm ENMs integration and concentration, in vitro and in vivo toxicity tests have been known to be highly inconsistent and sometimes do not agree with each other [54,55], possibly due to variation between cell culture lines and animal species [56] and issues with testing method accuracy [57]. The OECD has made several adjustments to their nanotoxicity testing protocols to combat this variation problem; however, to this day, no singular method has proven to be the golden standard for testing nanotoxicity in ENMs, and methods continue to be tested and enhanced/modified to keep up with the accelerating ENMs development field [58].

For example, the OECD has implemented newly revised inhalation toxicity testing guidelines, 412 and 413, for 28-day and 90-day inhalation toxicity studies, respectively, for carbon-based ENMs. These methods focus on inhalation since it is a primary route of ENMs exposure in humans [59], with employees of carbon-based ENMs manufacturing being the largest group at risk of physical contact. Due to an increase in the number of applicational fields, there is a growing concern about overall hazardous potential when it comes to inhalation [60]. However, according to a study performed by Kim et al., there is a significant lack of data on the toxicity of multi-walled carbon nanotubes (MWCNTs) [61,62]. Nonetheless, following the OECD guideline [63], Kim et al. conducted a 28-day inhalation toxicity study by exposing rats to MWCNTs at 0, 0.257, 1.439, and 4.253 mg/m^3^ for 28 days [61]. They generated their MWCNTs aerosols using an acoustic dry aerosol generator and tested the aerosol chamber concentrations using OC/EC and field emission-transmission electron microscope (FE-TEM) to confirm MWCNTs exposure. Cytotoxicity markers lactate dehydrogenase (LDH), micro-albumin (mALB), and micro-total protein (mTP) were examined in bronchoalveolar lavage fluid (BALF) for toxicity analysis as proposed by the OECD method. Samples of the rat lungs were taken after 1 day, 7 days, and 28 days post-exposure (PEO-1, PEO-7, and PEO-28). They noticed that the alveolar macrophages of the lungs contained MWCNTs material in all the samples. The low concentration (0.257 mg/m^3^) did not show any pneumocyte damage or cell inflammation. Agglomerated MWCNTs, however, were seen throughout the lungs, including the bronchi, alveolar ducts, and alveoli [61]. Additionally, the moderate and high concentration samples (1.439 and 4.253 mg/m^3^) showed granulomatous lesions filled with MWCNTs on all PEO sampling days. It is of note that Kim et al. did not find any significant organ weight changes after exposure for all time periods, demonstrating that solely relying on organ weight as an indication of toxicity is inadequate, which was also pointed out in the study by Weaver et al. However, toxicity results varied drastically between similar studies and studies that used different types of MWCNTs [29,64,65]. Although the new OECD method significantly improved the previous 412 method, the lack of data available on MWCNTs proved to be a major setback with general testing methods.

## 4. New and Enhanced Methods of Nanotoxicity Determination and Particle Detection

Although the above methods help detect ENMs and NPs toxicity, there remains an issue with the accuracy of the methods [57,66]. Part of the reason is due to the lack of technology applied to analytical methods. With the ever-advancing field of nanomaterials, analytical methods lag behind or suffer due to lack of data, as shown previously with the OECD 412 method. Additionally, if there are no measures taken to confirm the successful integration of the ENMs or NPs into the cells/organisms being tested for toxicity, there cannot be an accurate follow-up evaluation/conclusion that the concentration of ENMs/NPs injected caused toxicity. Furthermore, research articles do not necessarily expand beyond their target application for ENMs degradation and integration into other matrices and systems, particularly their environmental exposure/fate [67], leading to many published articles in the nanotoxicity field being limited and questionable [68]. In fact, most published ENMs test methods for environmental or biological testing applications have not been validated following the procedures set by the USEPA or other regulatory bodies [57]. Although the regulatory guidance for the testing of chemicals set by the USEPA, OECD, and AIHA provides extensive guidelines that help protect both humans and the environment, there remains limited guidance for analytical test methods or toxicity assessment procedures for direct measurements of ENMs, rather than indirect measurements [66]. This exemplifies the need to advance further analytical instrumentation and test methods to better directly, qualitatively and quantitatively evaluate ENMs, especially in complex biological and environmental matrices (e.g., air, sludge, and water) [69]. In many environmental and biological testing cases, there is a lack of sufficient standards to compare with real-time measurement analyses [70,71]. Ultimately, analytical methods need to be improved, or new methods must be developed to counter the detection and accuracy problems seen in nanotoxicity assessments [72].

Such advancements in both analytical instrumentation and test methods can be seen in several recently published articles. The first is by Mader et al., who applied the USEPA analytical test method validation guidance to develop a new test method for the quantitation of engineered NPs in water matrices [66]. The validated ENMs analytical test method for water is not limited to metal-containing NPs and was applied to two OECD ecotoxicity test methods for both Daphnia and algae; by direct measurement of nanoparticle size distribution and concentration in the ecotoxicity test matrix. Analytical NPs measurement was performed on a liquid nanoparticle sizer [73], differential mobility analyzer [74], and nano water-based condensation particle counter [75]. The role of the liquid nanoparticle sizer was crucial as it quantitatively diluted sample solutions using ultrapure water by a 20:1 to 20,000:1 ratio prior to nebulization. The nebulizer was adjusted to produce an aqueous aerosol with a droplet size of 300 nm, with the sample dilution ensuring that only one particle was present in each droplet. The resulting nebulized aerosol was then dried, classified and counted. The combination of the two instruments allowed for measuring the number of each size of particles in a volume of air. Additionally, the number-weighted NPs size distribution could be determined by scanning a range of particle mobility in the differential mobility analyzer. The validated Mader et al. method quantified both the NPs size distribution and dose level verification concentrations in the Daphnia and algae ecotoxicity test matrices. The most important factor in accurate quantitative measurements for the method was the application of matrix-matched NPs standard calibration curves to minimize analytical response factor differences between the standards and test matrices [63]. The analytical method requires the use of certified NPs reference materials for calibration standard preparations. Because of the availability of other certified metal and non-metal NPs materials, it is possible to adapt their EPS guidance validated test method for other ENMs and in other water matrices (e.g., drinking water, wastewater, groundwater) [66].

Additionally, Savić-Zdraković et al. [76] also utilized an OECD testing guideline, in this case, guideline 218 [77], to examine CeO_2_ NPs uptake in relation to oxidative stress parameters, in vivo genotoxic effects, larvae, and life-trait toxicity parameters using ICP-MS analysis. This study established the importance of establishing a standardized methodology for larvae lethality and sub-lethality cutoffs as their results indicated that the larvae were not at risk of CeO_2_ NPs toxicity; however, accumulation of these particles could impact organisms that consume the larvae. Therefore, much like the Kim et al. study, the value of the OECD guideline is greatly impacted by the lack of data surrounding CeO_2_ NPs toxicity testing, which also contributes to these NPs being listed on the OECD priority list of environmental impact assessment [76].

Another method that advanced the analytical side of NPs detection was presented by Hadioui et al., who discussed detecting NPs in the environment by inductively coupled plasma mass spectrometry (ICP-MS). This is one of the best analytical techniques for detecting ENMs [78,79]. In addition to ICP-MS being limited to metal-based NPs, it is also limited by particle size detection limits, with many NPs and their oxides being out of the detection range. Hadioui et al. helped improve this detection limit by adjusting the kind of aerosols introduced into the ICP-MS. They examined different nebulization desolvating techniques, distinguished as “dry” and “wet” aerosols. Hadioui et al. noted an increased number of counts in the dry mode, and for smaller particles (9 nm Ag), more ions were extracted using the desolvation for both wet and dry aerosols. Both desolvating systems lead to an increased signal intensity of the 9 nm Ag and 25 nm TiO_2_ NPs. Thus, dry aerosols had a better detection and resolved peak intensities for small NPs [78]. Additionally, an increase in sensitivity was also noted for the 5 nm Ag NPs. Injecting 2.3 ng/L, the dry aerosol compared to the wet aerosol showed a drastic increase in the detected particles; no particles were detected when using the ICP-Q-MS (the quadrupole ICP-MS used to evaluate instrument sensitivity) [77]. The size detection limit for ICP-Q-MS was 17 nm, while a single-particle sector field ICP-MS (ICP-SF-MS) was 5 nm, which could further be reduced to 3 nm using the desolvating nebulizer. Thus, by using dry aerosols for ICP-MS, Hadioui et al. successfully improved sensitivity and enhanced ion extraction [78].

Cui et al., in their study, helped examine the fate and improved detection of TiO_2_ NPs in the environment using ICP-MS by changing synthesis parameters, utilizing Ho as a chemical marker in their NPs and thus designing NaHoF_4_@TiO_2_ NPs. Using an Al(OH)_3_ layer around Ho in NaHoF_4_, the added colloidal stability and hydrophilic surface helped TiO_2_ deposition and coating when synthesizing the NPs [79]. The goal of the Cui et al. study was to be able to detect engineered TiO_2_ NPs in the environment without Ti background interference. Using their unique synthesis, the addition of Ho as a tracer significantly helped detect the engineering TiO_2_ NPs in the environment, despite being in low concentrations (100 million-fold dilutions or 5000–200,000 particles/mL) [80].

As for the biological fates of nanoparticles, studies such as Turco et al. [81] and López-Serrano Oliver et al. [82] provide valuable insights into biological nanotoxicity testing. Turco et al. utilized a sputtering-enabled intracellular X-ray photoelectron spectroscopy (SEI-XPS) method in which metallic NPs were cultured in media and cells before being directly measured for their internalization, stability, and oxidation state. Utilizing this technique, Turco et al. provided a possible method to help assess NPs integration, accumulation, and longevity and thus provide valuable insight into nanotoxicity. López-Serrano Oliver et al. also looked at metal-based nanoparticles, in the form of silver, and focused on developing a new mass cytometry method that can quantify NPs numbers per single cell [82]. Although they made some interesting and important discoveries for new nanotoxicity analyses of NPs, there remains an issue with monitoring and intracellular uptake of NPs out of the mass range of mass cytometers.

Lastly, a fairly recent method to determine nanotoxicity is through in silico analyses. These methods have proven advantageous as they bypass the costs of extensive in vitro and in vivo experiments and remove the need for animal experiments [83]. These in silico studies helped provide insight into potential alternative testing methods for nanotoxicity as existing experimental data surrounding the tested NPs and ENMs confirmed the results of the computational data obtained [84,85]. However, the main issue with in silico analyses is similar to the in vitro and in vivo experiments, which is a lack and inconsistency of data. In silico methods are dependent on existing nanotoxicity data to confirm the model’s accuracy [86]. Therefore, the future accuracy of in silico analysis is also dependent on advancing current analytical methods to better analyze nanotoxicity experimentally.

Through all of these studies, it is seen that analytical methods and the increased availability of certified analytical NPs standards are vital in the measurement of ENMs and NPs in toxicity assessments as these materials need to be accurately identified and quantified in both biological and environmental matrices [87]. Although progress has been made in developing toxicity assessments and testing methods, the accuracy of these methods has been called into question on more than one occasion [88]. Agencies like OECD and AIHA help provide guidelines for nanotoxicity testing. However, a repeated problem across studies is the lack of data and certified analytical standards surrounding NPs and ENMs, particularly the newer ones that are being produced at a rate that is not matched by the development of the analytical instrumentation, even with enhanced methods and new analytical techniques [89]. There is also the problem with long-term ENMs and NPs exposure/integration, with toxic degradation particles potentially never being assessed as NPs and ENMs production continues. The generational and long-term biological and environmental impacts of ENMs and NPs nanotoxicity is an underdeveloped field but one that should be considered for exploration [90].

## 5. Conclusions

When it comes to the world of nanoscience, its vast diversity in engineered materials makes this area of research rich in possibility. Still, it ultimately leads to uncertainty in the health of humans and the environment they come into contact with. Truly, nanotoxicity data are abundant among research articles, but small groups of ENMs and NPs tend to be studied, and their long-term environmental fate or chemical/physical changes over time are not analyzed [91]. Even with several agencies and organizations across multiple countries pooling their information together on the topic of nanotoxicity safety policies [92], the problem remains; as the field of nanoscience advances, analytical instrumentation and methods struggle to find applicability when it comes to accurately measuring ENMs’ and NPs’ harmful effects and possible integration into alternate media. Although traditional biological testing such as in vitro and in vivo methods help glimpse the impact NPs might have on chosen cells and living organisms, these methods tend to be indirect measurements of ENMs by analyzing organ weight, mitochondrial function, and ROS levels. Indirect methods tend to only analyze the cells’ or organism’s response to the ENMs without confirming the exact amounts of ENMs involved in the negative or positive response to the ENMs. As a result, there has been a movement to develop direct methods of measuring ENMs in the environment and biological systems. It remains a challenge for multiple national and international agencies to assess the toxicity of ENMs accurately. As new NPs and ENMs are synthesized, researchers outside of the agencies that designed them have amended many existing protocols and methods for their detection, still with no one method being suitable for all ENMs or NPs. A significant limitation with the development of nanomaterials is that traditional regulatory and scientific methods used to assess the biological and environmental toxicity of chemicals do not generally apply to the assessment of nanomaterials. This limitation is directly related to the need for advancements and further developments in analytical instrumentation, analytical test methods, and analytical standards for directly measuring ENMs and NPs in biological and environmental matrices.

There is no doubt that NPs and ENMs pose potential risks environmentally and biologically [93]. Without addressing the issues of accurate and consistent nanotoxicity determination, unknown degradation products, accumulation, and induced toxicity are of increasing concern. Given that ENMs and NPs are continually being advanced and incorporated into commercial products and medical treatments, the potential risk of undetected toxicity in the short- or long-term is also increasing. Particularly for the use of NPs in medicine, it is of utmost importance that treatment NPs are precisely characterized, detected and traceable, that their degradation products are not harmful, and that short- and long-term toxicity is not induced in the treated patient, especially for human trials. Similarly, the environmental fate of NPs and ENMs needs to be heavily considered as they can accumulate in the soil, water, plants, and animals. If not properly detected, quantified, and removed as contaminants, NPs and ENMs, both whole or degraded, can pose increasing environmental risk and potentially cause irreversible harm to the ecosystem. With 9806 products [94] currently incorporating nanomaterials, it is vital to address the problem of detection limits and improve analytical testing methods.

## Figures and Tables

**Table 1:** In vitro assay types used for the analysis of nanotoxicity.

Assay Type	Cell Toxicity Investigation
Proliferation	Cell metabolism
Apoptosis	DNA, protein, and lipid damage
Necrosis	Membrane integrity
Oxidative Stress	DNA/RNA damage, lipid peroxidation, protein oxidation/nitration, ROS generation, antioxidant counterbalance

**Table 2:** In vivo methods for nanotoxicity determination.

Methods	Toxicity Analysis Examination Types
Radiolabeling	Radioisotope tracking of NPs through biological systems examined for biodistribution
Clearance	Excretion and metabolism of NPs examined after various exposure times
Serum Chemistry	Enzymes, lipids, hormones, and proteins in serum examined for metabolic interferences
Histopathology	Cells, tissues, and organs examined for disease manifestation
Hematology	Red and white blood cells, platelets, and coagulation system examined for disorders

## References

[1] National Nanotechnology Initiative. https://www.nano.gov/you/nanotechnology-benefits.

[2] Lowry G., Gregory K., Apte S., Lead J. (2012). Transformations of nanomaterials in the environment. Environ. Sci. Technol..

[3] Nanobiomedical Centre. http://cnbm.amu.edu.pl/en/nanomaterials.

[4] Gupta R., Xie H. (2018). Nanoparticles in daily life: Applications, toxicity, and regulations. J. Environ. Pathol. Toxicol. Oncol..

[5] Lehman S., Morris A., Mueller P., Salem A., Grassian V., Larsen S. (2016). Silica nanoparticle-generated ROS as a predictor of cellular toxicity: Mechanistic insights and safety by design. Environ. Sci. Nano.

[6] Mugica I., Fito C., Domat M., Dohányosová P., Gutierrez-Cañas C., López-Videl S. (2017). Novel techniques for detection and characterization of nanomaterials based on aerosol science supporting environmental applications. Sci. Total Environ..

[7] Qiu T., Clement P., Haynes C. (2018). Linking nanomaterial properties to biological outcomes: Analytical chemistry challenges in nanotoxicology for the next decade. Chem. Commun..

[8] Pietroiusti A., Stockmann-Juvala H., Lacaroni F., Savolainen K. (2018). Nanomaterial exposure, toxicity, and impact on human health. Wiley Interdiscip. Rev. Nanomed. Nanobiotechnology.

[9] Ganguly P., Breen A., Pillai S. (2018). Toxicity of nanomaterials: Exposure, pathways, assessment, and recent advances. ACS Biomater. Sci. Eng..

[10] Jahan S., Yusoff I., Alias Y., Bakar A. (2017). Reviews of the toxicity behavior of five potential engineered nanomaterials (ENMs) into the aquatic ecosystem. Toxicol. Rep..

[11] Saleh T. (2020). Trends in the sample preparation and analysis of nanomaterials as environmental contaminants. Trends Environ. Anal. Chem..

[12] Peters R., van Bemmel G., Milani N., den Hertog G., Undas A., van der Lee M., Bouwmeester H. (2018). Detection of nanoparticles in Dutch surface waters. Sci. Total Environ..

[13] Dayem A., Hossain M., Lee S., Kim K., Saha S., Yang G., Choi H., Cho S. (2017). The role of reactive oxygen species (ROS) in the biological activities of metallic nanoparticles. Int. J. Mol. Sci..

[14] Yu Z., Li Q., Wang J., Yu Y., Wang Y., Zhou Q., Li P. (2020). Reactive oxygen spcies-related nanoparticle toxicity in the biomedical field. Nanoscale Res. Lett..

[15] Perillo B., Di Donato M., Pezone A., Di Zazzo E., Giovannelli P., Galasso G., Castoria G., Migliaccio A. (2020). ROS in cancer therapy: The bright side of the moon. Exp. Mol. Med..

[16] Liou G., Storz P. (2010). Reactive oxygen species in cancer. Free Radic. Res..

[17] Aggarwal V., Tuli H.S., Varol A., Thakral F., Yerer M.B., Sak K., Varol M., Jain A., Khan M.A., Sethi G. (2019). Role of reactive oxygen species in cancer progression: Molecular mechanisms and recent advancements. Biomolecules.

[18] OECD. https://www.oecd.org/chemicalsafety/nanosafety/overview-testing-programme-manufactured-nanomaterials.htm.

[19] Savage D., Hilt J., Dziubla T. (2019). In vitro methods for assessing nanoparticle toxicity. Nanotoxicity.

[20] Balke J., Volz P., Neumann F., Brodwolf R., Wolf A., Pischon H., Radbruch M., Mundhenk L., Gruber A., Ma N. (2018). Visualizing oxidative cellular stress induced by nanoparticles in the subcytotoxic range using fluorescence lifetime imaging. Small.

[21] Wu L., Sedgwick A., Sun X., Bull S., He X., James T. (2019). Reactive-based fluorescent probes for the detection and imaging of reactive oxygen, nitrogen, and sulfur species. Acc. Chem. Res..

[22] Dai Y., Yang Z., Cheng S., Wang Z., Zhang R., Zhu G., Wang Z., Yung B., Tian R., Jacobson O. (2018). Toxic reactive oxygen species enhanced synergistic combination therapy by self-assembled metal-phenolic network nanoparticles. Adv. Mater..

[23] Kumar V., Sharma N., Maitra S. (2017). In vitro and in vivo toxicity assessment of nanoparticles. Int. Nano Lett..

[24] Dönmez Güngüneş Ç., Şeker Ş., Elçin A., Elçin Y. (2017). A comparative study on the in vitro cytotoxic responses of two mammalian cell types to fullerenes, carbon nanotubes and iron oxide nanoparticles. Drug Chem. Toxicol..

[25] Li N., Chen L., Zeng C., Yang H., He S., Wei Q. (2021). Comparative toxicity, biodistribution and excretion of ultra-small gold nanoclusters with different emission wavelengths. J. Biomed. Nanotechnol..

[26] Gao Y., Wu W., Qiao K., Feng J., Zhu L., Zhu X. (2021). Bioavailability and toxicity of silver nanoparticles: Determination based on toxicokinetic-toxicodynamic processes. Water Res..

[27] Zhang Y., Poon W., Tavares A., McGilvray I., Chan W. (2016). Nanoparticle-liver interactions: Cellular uptake and hepatobiliary elimination. J. Control. Release.

[28] Pritchard N., Kaitu’u-Lino T., Harris L., Tong S., Hannan N. (2021). Nanoparticles in pregnancy: The next frontier in reproductive therapeutics. Hum. Reprod. Update.

[29] Báez D., Gallardo-Toledo E., Paz Oyarzún M., Araya E., Kogan M. (2021). The influence of size and chemical composition of silver and gold nanoparticles on in vivo toxicity with potential applications to central nervous system diseases. Int. J. Nanomed..

[30] Strojny B., Kurantowicz N., Sawosz E., Grodzik M., Jaworski S., Kutwin M., Wierzbicki M., Hotowy A., Lipińska L., Chwalibog A. (2015). Long term influence of carbon nanoparticles on health and liver status in rats. PLoS ONE.

[31] Zhang M., Bishop B., Thompson N., Hildahl K., Dang B., Mironchuk O., Chen N., Aoki R., Holmberg V., Nance E. (2019). Quantum dot cellular uptake and toxicity in the developing brain: Implications for use as imaging probes. Nanoscale Adv..

[32] Xie J., Shen Z., Anraku Y., Kataoka K., Chen X. (2019). Nanomaterial-based blood-brain-barrier (BBB) crossing strategies. Biomaterials.

[33] Lee D., Minko T. (2021). Nanotherapeutics for nose-to-brain drug dlievery: An approach to bypass the blood brain barrier. Pharmaceutics.

[34] Chowdhry A., Kaur J., Khatri M., Puri V., Tuli R., Puri S. (2019). Characterization of functionalized multiwalled carbon nanotubes and comparison of their cellular toxicity between HEK 293 cells and zebra fish in vivo. Heliyon.

[35] Marisa I., Asnicar D., Matozzo V., Martucci A., Finos L., Marin M. (2021). Toxicological effects and bioaccumulation of fullerene C_60_ (FC_60_) in the marine bivalve *Ruditapes philippinarum*. Ecotoxicol. Environ. Saf..

[36] Tian J., Li J., Yin H., Ma L., Zhang J., Zhai Q., Duan S., Zhang L. (2021). In vitro and in vivo uterine metabolic disorders induced by silica nanoparticles through the AMPK signaling pathway. Sci. Total Environ..

[37] Lindén M. (2018). Biodistribution and excretion of intravenously injected mesoporous silica nanoparticles: Implications for drug delivery efficiency and safety. Enzymes.

[38] Bartucci R., Paramanandana A., Boersma Y., Olinga P., Salvati A. (2020). Comparative study of nanoparticle uptake and impact in murine lung, liver and kidney tissue slices. Nanotoxicology.

[39] Waegeneers N., Brasseur A., Van Doren E., Van der Heyden S., Serreyn P., Pussemier L., Mast J., Schneider Y., Ruttens A., Roels S. (2018). Short-term biodistribution and clearance of intravenously administered silica nanoparticles. Toxicol. Res..

[40] Mohammadpour R., Yazdimamaghani M., Cheney D., Jedrzkiewicz J., Ghandehari H. (2019). Subchronic toxicity of silica nanoparticles as a function of size and porosity. J. Control. Release.

[41] Huang Y., Cambre M., Lee H. (2017). The toxicity of nanoparticles depends on multiple molecular and physicochemical mechanisms. Int. J. Mol. Sci..

[42] Zhao X., Wang Y., Ji Y., Mei R., Chen Y., Zhang Z., Wang X., Chen L. (2022). Polystyrene nanoplastics demonstrate high structural stability in vivo: A comparative study with silica nanoparticles via SERS tag labeling. Chemosphere.

[43] Rana A., Yadav K., Jagadevan S. (2020). A comprehensive review on green synthesis of nature-inspired metal nanoparticles: Mechanism, application and toxicity. J. Clean. Prod..

[44] Rosário F., Duarte I., Pinto R., Santos C., Hoet P., Oliveira H. (2020). Biodistribution and pulmonary metabolic effects of silver nanoparticles in mice following acute intratracheal instillations. Environ. Sci. Pollut. Res..

[45] Zhang Y., Liu A., Cornejo Y., Van Haute D., Berlin J. (2020). A systematic comparison of in vitro cell uptake and in vivo biodistribution for three classes of gold nanoparticles with saturated PEG coatings. PLoS ONE.

[46] Janßen H., Angrisani N., Kalies S., Hansmann F., Kietzmann M., Warwas D., Behrens P., Reifenrath J. (2020). Biodistribution, biocompatibility and targeted accumulation of magnetic nanoporous silica nanoparticles as drug carrier in orthopedics. J. Nanobiotechnol..

[47] Mohammadpour R., Cheney D., Grunberger J., Yazdimamaghani M., Jedrzkiewicz J., Isaacson K., Dobrovolskaia M., Ghandehari H. (2020). One-year chronic toxicity evaluation of single dose intravenously administered silica nanoparticles in mice and their *Ex vivo* human hemocompatibility. J. Control. Release.

[48] Chrishtop V., Prilepskii A., Nikonorova V., Mironov V. (2021). Nanosafety vs nanotoxicology: Adequate animal models for testing in vivo toxicity of nanoparticles. Toxicology.

[49] Weaver J., Tobin G., Ingle T., Bancos S., Stevens D., Rouse R., Howard K., Goodwin D., Knapton A., Li X. (2017). Evaluating the potential of gold, silver, and silica nanoparticles to saturate mononuclear phagocytic system tissues under repeat dosing conditions. Part. Fibre Toxicol..

[50] Pu K., Chattopadhyay N., Rao J. (2016). Recent advances of semiconducting polymer nanoparticles in *in vivo* molecular imaging. J. Control. Release.

[51] Voigt N., Henrich-Noack P., Kockentiedt S., Hintz W., Tomas J., Sabel B.A. (2014). Toxicity of polymeric nanoparticles in vivo and in vitro. J. Nanoparticle Res..

[52] NIST. https://www.nist.gov/news-events/news/2019/08/solving-big-problem-measuring-tiny-nanoparticles.

[53] Shang J., Gao X. (2014). Nanoparticle counting: Towards accurate determination of the molar concentration. Chem. Soc. Rev..

[54] Sayes C., Reed K., Warheit D. (2007). Assessing toxicity of fine and nanoparticles: Comparing in vitro measurements to in vivo pulmonary toxicity profiles. Toxicol. Sci..

[55] Fischer H., Chan W. (2007). Nanotoxicity: The growing need for in vivo study. Curr. Opin. Biotechnol..

[56] Jesus S., Schmutz M., Som C., Borchard G., Wick P., Borges O. (2019). Hazard assessment of polymeric nanobiomaterials for drug delivery: What can we learn from literature so far. Bioeng. Biotechnol..

[57] Hund-Rinke K., Baun A., Cupi D., Fernandes T., Handy R., Kinross J., Navas J., Peijnenburg W., Schlich K., Shaw B. (2016). Regulatory ecotoxicity testing of nanomaterials—Proposed modifications of OECD test guidelines based on laboratory experience with silver and titanium dioxide nanoparticles. Nanotoxicology.

[58] Rasmussen K., Gonzalez M., Kearns P., Riego Sintes J., Rossi F., Sayre P. (2016). Review of achievements of the OECD working party on manufactured nanomaterials’ testing and assessment programme: From exploratory testing to testing guidelines. Regul. Toxicol. Pharmacol..

[59] Oberbek P., Kozikowski P., Czarnecka K., Sobiech P., Jakubiak S., Jankowski T. (2019). Inhalation exposure to various nanoparticles in work environment—Contextual information and results of measurements. J. Nanoparticle Res..

[60] Felley-Bosco E., MacFarlane M. (2018). Asbestos: Modern insights for toxicology in the era of engineered nanomaterials. Chem. Res. Toxicol..

[61] Kim J., Jo M., Kim Y., Kim T., Shin J., Kim B., Kim H., Lee H., Kim H., Ahn K. (2019). 28-Day inhalation toxicity study with evolution of lung deposition and retention of tangled multi-walled carbon nanotubes. Nanotoxicology.

[62] Fujita K., Obara S., Maru J., Endoh S. (2020). Cytotoxicity profiles of multi-walled carbon nanotubes with different physico-chemical properties. Toxicol. Mech. Methods.

[63] OECD. https://www.oecd.org/env/ehs/testing/test-no-412-subacute-inhalation-toxicity-28-day-study-9789264070783-en.htm.

[64] Nerl H., Cheng C., Goode A., Bergin S., Lich B., Gass M., Porter A. (2011). Imaging methods for determining uptake ad toxicity of carbon nanotubes in vitro and in vivo. Nanomedicine.

[65] Lama S., Merlin-Zhang O., Yang C. (2020). In vitro and in vivo models for evaluating the oral toxicity of nanomedicines. Nanomaterials.

[66] Mader B., Ellefson M., Wolf S. (2015). Measurements of nanomaterials in environmentally relevant water matrices using liquid nebulization/differential mobility analysis. Environ. Toxicol. Chem..

[67] Barbosa F., Adeyemi J., Bocato M., Comas A., Campiglia A. (2020). A critical viewpoint of current issue, limitations, and future research needs on micro- and nanoplastic studies: From the detection to the toxicological assessment. Environ. Res..

[68] Krug H.F. (2018). The uncertainty with nanosafety: Validity and reliability of published data. Colloids Surf. B Biointerfaces.

[69] AIHA. https://aiha-assets.sfo2.digitaloceanspaces.com/AIHA/resources/Nanoparticle-Sampling-and-Analysis-Fact-Sheet.pdf.

[70] Desai N. (2012). Challenges in development of nanoparticle-based therapeutics. AAPS J..

[71] Sahu S., Hayes A. (2017). Toxicity of nanomaterials found in human environment: A literature review. Toxicol. Res. Appl..

[72] Lead J., Batley G., Alvarez P., Croteau M., Handy R., McLaughlin M., Judy J., Schirmer K. (2018). Nanomaterials in the environment: Behavior, fate, bioavailability, and effects—an updated review. Environ. Toxicol. Chem..

[73] TSI. https://tsi.com/products/particle-sizers/particle-size-spectrometers/.

[74] TSI. https://tsi.com/products/particle-sizers/electrostatic-classifiers-and-dmas/differential-mobility-analyzer-3081/.

[75] TSI. https://tsi.com/discontinued-products/nano-water-based-condensation-particle-counter-.

[76] Savić-Zdraković D., Milošević D., Uluer E., Duran H., Matić S., Stanić S., Vidmar J., Šćanćar J., Dikic D., Jovanović B. (2020). A multiparametric approach to cerium oxide nanoparticle toxicity assessment in non-biting midges. Environ. Toxicol. Chem..

[77] OECD. https://www.oecd-ilibrary.org/environment/test-no-218-sediment-water-chironomid-toxicity-using-spiked-sediment_9789264070264-en.

[78] Hadioui M., Knapp G., Azimzada A., Jreije I., Frechette-Viens L., Wilkinson K. (2019). Lowering the size detection limits of Ag and TiO2 nanoparticles by single particle ICP-MS. Anal. Chem..

[79] Tharaud M., Louvat P., Benedetti M. (2021). Detection of nanoparticles by single-particle ICP-MS with complete transport efficiency through direct nebulization at few-microliters-per-minute uptake rates. Anal. Bioanal. Chem..

[80] Cui X., Fryer B., Zhou D., Lodge R., Khlobystov A., Valsami-Jones E., Lynch I. (2019). Core-shell NaHoF_4_@TiO_2_ NPs: A labeling method to trace engineered nanomaterials of ubiquitous elements in the environment. ACS Appl. Mater. Interfaces.

[81] Turco A., Moglianetti M., Corvaglia S., Rella S., Catelani T., Marotta R., Malitesta C., Pompa P. (2018). Sputtering-enabled intracellular X-ray photoelectron spectroscopy: A versatile method to analyze the biological fate of metal nanoparticles. ACS Nano.

[82] López-Serrano Oliver A., Haase A., Peddinghaus A., Wittke D., Jakubowski N., Luch A., Grützkau A., Baumgart S. (2019). Mass cytometry enabling absolute and fast quantification of silver nanoparticle uptake at the single cell level. Anal. Chem..

[83] Forest V. (2022). Experimental and computational nanotoxicology—Complementary approaches for nanomaterial hazard assessment. Nanomaterials.

[84] Tsukanov A., Turk B., Vasiljeva O., Psakhie S. (2022). Computational indicator approach for the assessment of nanotoxicity of two-dimensional nanomaterials. Nanomaterials.

[85] Huang H., Lee Y., Hsu Y., Liao C., Lin Y., Chiu H. (2021). Current strategies in assessment of nanotoxicity: Alternatives to in vivo animal testing. Int. J. Mol. Sci..

[86] Taka A., Tata C., Klink M., Mbianda X., Mtunzi F., Naidoo E. (2021). A review on conventional and advanced methods for nanotoxicology evaluation of engineered nanomaterials. Molecules.

[87] Monikh F., Chupani L., Vijver M., Vancová M., Peijnenburg W. (2019). Analytical approaches for characterizing and quantifying engineered nanoparticles in biological matrices from an (eco)toxicological perspective: Old challenges, new methods, and techniques. Sci. Total Environ..

[88] Arvidsson R., Baun A., Furberg A., Hansen S., Molander S. (2018). Proxy measures for simplified environmental assessment of manufactured nanomaterials. Environ. Sci. Technol..

[89] Fakhrullin R., Nigamatxyanova L., Fakhrullina G. (2021). Dark field/hyperspectral microscopy for detecting nanoscale particles in environmental nanotoxicology research. Sci. Total Environ..

[90] Ellis L., Valsami-Jones E., Lynch I. (2020). Exposure medium and particle ageing moderate the toxicological effects of nanomaterials to Daphnia magna over multiple generations: A case for standard test review?. Environ. Sci. Nano.

[91] Wigger H., Kägi R., Wiesner M., Nowack B. (2020). Exposure and possible risks of engineered nanomaterials in the environment—Current knowledge and directions for the future. Rev. Geophys..

[92] Demirbaş K., Çevik S. (2020). Regulatory policies for safety of nanomaterials. Open J. Nano.

[93] Chugh G., Siddique K., Solaiman Z. (2021). Nanobiotechnology for agriculture: Smart technology for combating nutrient deficiencies with nanotoxicity challenges. Sustainability.

[94] Nanotechnology Products Database. https://product.statnano.com/.

